# The impact of co-morbidity on the quality of life of people with dementia: findings from the IDEAL study

**DOI:** 10.1093/ageing/afy155

**Published:** 2018-11-07

**Authors:** Sharon M Nelis, Yu-Tzu Wu, Fiona E Matthews, Anthony Martyr, Catherine Quinn, Isla Rippon, Jennifer Rusted, Jeanette M Thom, Michael D Kopelman, John V Hindle, Roy W Jones, Linda Clare

**Affiliations:** 1 REACH: The Centre for Research in Ageing and Cognitive Health, University of Exeter Medical School and College of Life and Environmental Sciences, St Luke’s Campus, Exeter, UK; 2 King’s College London, Social Epidemiology Research Group, Health Service and Population Research, Institute of Psychiatry, Psychology & Neuroscience, London, UK; 3 Institute of Health and Society, Newcastle University, Newcastle Upon Tyne, UK; 4 College of Health and Life Sciences, Brunel University London, London, UK; 5 School of Psychology, University of Sussex, Brighton, UK; 6 School of Medical Sciences, University of New South Wales, Sydney, Australia; 7 King’s College London, Psychological Medicine, Institute of Psychiatry Psychology and Neuroscience, UK; 8 RICE (The Research Institute for the Care of Older People), Bath, UK; 9 Wellcome Centre for Cultures and Environments of Health, University of Exeter, Exeter, UK

**Keywords:** Alzheimer’s, multimorbidities, depression, older people

## Abstract

**Background:**

The aim was to investigate the co-morbidity profile of people with dementia and examine the associations between severity of co-morbidity, health-related quality of life (HRQoL) and quality of life (QoL).

**Methods:**

The improving the experience of Dementia and Enhancing Active Life (IDEAL) cohort consisted of 1,547 people diagnosed with dementia who provided information on the number and type of co-morbid conditions. Participants also provided ratings of their health-related and dementia-specific QoL.

**Results:**

The majority of the sample were living with more than one chronic condition. Hypertension was commonly reported and frequently combined with connective tissue disease, diabetes and depression. The number of co-morbid conditions was associated with low QoL scores, and those with severe co-morbidity (≥5 conditions) showed the greatest impact on their well-being.

**Conclusions:**

Co-morbidity is an important risk factor for poor QoL and health status in people with dementia. Greater recognition of the nature and impact of co-morbidity is needed to inform support and interventions for people with dementia and a multidisciplinary approach to care provision is recommended.

## Key points

- The majority of people with dementia were living with one or two chronic health conditions, and hypertension was the most frequent co-morbid condition.
- Multiple health conditions have important consequences for the quality of life (QoL) and well-being of the individual.
- This study highlights the need for better care planning and the organisation of care provision to deal with multiple conditions in an integrated way.

## 

Living with dementia poses many challenges for people with dementia and for those who care for them. This may be coupled with other health problems as the accumulation of changes associated with ageing can lead to the accrual of illnesses and disabilities [1]. With the increased prevalence of individual health conditions associated with ageing [2], there is growing interest in the co-occurrence of medical conditions and the implications for the individual. Multimorbidity and co-morbidity are used interchangeably to describe the presence of co-occurring diseases but definitions vary across studies. Multimorbidity is the co-occurrence of two or more diseases or active health conditions that may or may not be linked by a causal relationship or with no consistent dominant index disorder. Co-morbidity is the term given to the presence of conditions existing concurrently with a condition considered as the primary or index disease [1] and this study will focus on the presence of conditions in addition to dementia as the index condition. Co-morbidities significantly associated with dementia include physical health conditions such as congestive cerebrovascular disease, and cardiac arrhythmia [3], hypertension and diabetes [4], and depression [5].

People with dementia are more likely to have five or more health conditions and more prescription usage than those without dementia [6], and co-morbidity ranging from two to eight conditions has been reported [7]. Examination of the medical records in England showed that 92% of people with dementia had a formal diagnosis of at least one other disease, and 53% were considered to have co-morbidity with three or more conditions [2]. Similarly, 61% of people with Alzheimer’s disease across various care settings had three or more conditions [8]. Co-morbidity rates were the same for people with dementia compared with those without dementia in a primary care [7], but for care home residents, rates were higher in people with dementia compared with older care residents without dementia [9]. There is growing interest not only in the number of co-morbid conditions but also in the combinations or array of conditions referred to as ‘morbidotypes’ [10].

Co-morbidity can influence a range of health outcomes such as mortality [11] and disability [12]. Multimorbidity is negatively associated with health-related quality of life (HRQoL) [9, 13], and certain disease combinations, such as diabetes and coronary disorders, impact more on HRQoL than others [14]. Higher co-morbidity in people with dementia is negatively associated with ratings of disease-specific QoL [15]. Generally there is a negative association between co-morbidity and QoL but the results are not conclusive, with variable findings related to QoL and HRQoL [16], and few studies examining both perceptions of health-related QoL and well-being.

People with dementia are living with co-morbidity, and greater recognition of the extent and impact of this burden of disease is essential for the identification of interventions to help maintain independence and improve QOL [17]. Co-morbidity poses significant challenges for care provision, and greater coordination of care planning across conditions is needed [4, 18]. The aim of this study is to investigate the co-morbidity profile of people with dementia and to examine the associations between severity of co-morbidity, HRQoL and QoL using a large community-based cohort of people with dementia.

## Method

### Sample

Participants were drawn from the baseline wave of the ‘Improving the experience of Dementia and Enhancing Active Life’ (IDEAL) longitudinal cohort study recruited across Great Britain [19]. Inclusion criteria included a clinical diagnosis of dementia and a Mini-Mental State Examination (MMSE) score ≥15 [20]. Interviews were conducted in participant homes. Analysis is based on V2 of the IDEAL baseline (T1) dataset.

### Ethics statement

Written informed consent was secured for all participants. Ethics approval was granted by the Wales Research Ethics Committee 5 (reference 13/WA/0405) and the Ethics Committee of the School of Psychology, Bangor University (reference 2014–11684). The IDEAL study is registered with UKCRN, registration number 16593.

### Measures

Information on the age profile, diagnostic subtypes and educational level of the cohort was included in analyses.

### Co-morbidity

A record of the presence or absence of 23 chronic conditions was collected using the Charlson co-morbidity index (CCI) [21], administered through a joint interview with the person with dementia and carer where available. The CCI included diseases selected on the basis of their association with mortality and this version of the CCI includes depression, hypertension, ulcers and use of warfarin [21]. A count of the diseases within the index is used as a measure of co-morbidity, and as we were interested in the impact of diseases in addition to our index condition dementia was not counted in the analysis. Co-morbidity was categorised into four levels of severity: no co-morbidity, mild (1–2 conditions), moderate (3–4 conditions) and severe (≥5 conditions).

### Health-related quality of life

Participants rated their HRQoL using the two parts of the EQ-5D-3L [22]:

- The EQ-5D-3L descriptive system has five dimensions: mobility, self-care, usual activities, pain/discomfort and anxiety/depression. The EQ-5D dimensions were dichotomised into ‘no problems’ and ‘moderate/severe problems’ for logistic regression analyses.
- The EQ-5D visual analogue scale (EQ-5D VAS) provides a self-rating of health on a vertical visual analogue scale from 0 = ‘worst imaginable health state’ to 100 = ‘best imaginable health state’. This is used as a quantitative measure of HRQoL.

### Quality of life

The QoL in Alzheimer’s disease (QoL-AD) scale was developed specifically for people with dementia and focuses on QoL domains important in cognitively impaired older people. Participants rate aspects of their current situation such as mood, memory and make a global assessment of QoL as a whole [23]. Scale scores range from 13 to 52, with higher scores representing greater QoL.

### Statistical analyses

The analysis first investigated the frequencies and prevalence of different conditions and their combinations. Linear regression was conducted to investigate the associations between severity of co-morbidity and both HRQoL and QoL-AD. The associations between severity of co-morbidity and the five individual dimensions of the EQ-5D were examined using logistic regression. The models were adjusted for participants’ sociodemographic characteristics including age, gender, education and dementia subtype.

## Results

The IDEAL cohort consisted of 1,547 people diagnosed with dementia at baseline; 56.3% were males. The mean MMSE score was 24.15 (s.d. 3.46; range 14–30). The mean age of the people with dementia was 76.4 (s.d. 8.5). The majority of the sample had a diagnosis of Alzheimer’s disease (55.5%). Characteristics of the sample are presented in Table 1.

**Table 1.** Demographic characteristics of the sample and level of co-morbid conditions

Characteristics	*N* (%)	Co-morbid conditions, *N* (%), missing (*n* = 85)			
		0	1–2	3–4	≥5
		None	Mild	Moderate	Severe
*N*	1,547	365 (25)	757 (52)	259 (17)	81 (5)
Age groups					
<65	136 (8.8)	48 (13.2)	60 (7.9)	14 (5.4)	10 (12.3)
65–69	178 (11.5)	44 (12.0)	95 (12.5)	23 (8.8)	10 (12.3)
70–74	260 (16.8)	70 (19.2)	122 (16.2)	39 (15.0)	13 (16.1)
75–79	370 (23.9)	83 (22.7)	184 (24.3)	62 (23.9)	21 (25.9)
80+	603 (38.9)	120 (32.9)	296 (39.1)	122 (46.7)	27 (33.3)
Sex					
Male	872 (56.3)	198 (54.2)	430 (56.8)	143 (55.2)	54 (66.6)
Female	675 (43.6)	167 (45.7)	327 (43.2)	116 (44.7)	27 (33.3)
Dementia diagnosis					
Alzheimer’s disease	858 (55.5)	246 (67.4)	435 (57.5)	108 (41.7)	34 (41.9)
Vascular dementia	171 (11.0)	21 (5.7)	64 (8.5)	56 (21.6)	18 (22.2)
Mixed (Alzheimer’s and vascular)	326 (21.0)	45 (12.2)	161 (21.3)	73 (28.2)	25 (30.8)
Frontotemporal dementia	54 (3.5)	20 (5.5)	26 (3.4)	6 (2.3)	0 (0.0)
Parkinson’s disease dementia	44 (2.8)	8 (2.2)	25 (3.3)	4 (1.5)	2 (2.5)
Lewy body dementia	53 (3.4)	15 (4.1)	26 (3.4)	7 (2.7)	2 (2.7)
Unspecified/Other	41 (2.7)	10 (2.7)	20 (2.6)	2 (2.5)	0 (0.0)
Quality of life measures					
QoL-AD total score, mean (SD)	36.7 (5.9)	38.6 (5.7)	37.1 (5.6)	34.5 (5.8)	32.7 (5.4)
EQ-5D VAS, mean (SD)	71.9 (18.6)	77.7 (16.7)	72.5 (17.8)	66.5 (19.3)	59.9 (21.5)
EQ-5D problems, *N* (%)					
Mobility					
None	897 (58.1)	280 (77.1)	458 (60.6)	101 (39.0)	14 (17.3)
Moderate/Severe	646 (41.8)	83 (22.8)	297 (39.3)	158 (61.0)	67 (82.7)
Self-care					
None	1,209 (82.8)	331 (90.9)	643 (84.2)	187 (72.0)	48 (59.2)
Moderate/Severe	252 (17.2)	33 (9.1)	114 (15.6)	72 (27.8)	33 (40.7)
Usual activities					
None	1,018 (66.1)	277 (76.3)	532 (70.6)	140 (54.2)	27 (33.3)
Moderate/Severe	522 (33.9)	86 (23.7)	221 (29.3)	118 (45.7)	54 (66.6)
Pain/Discomfort					
None	924 (59.8)	276 (75.8)	456 (60.3)	120 (46.5)	20 (24.7)
Moderate/Severe	620 (40.2)	88 (24.2)	300 (39.7)	138 (53.4)	61 (75.3)
Anxiety/Depression					
None	1,014 (65.8)	260 (71.4)	518 (68.7)	144 (56.0)	38 (46.9)
Moderate/Severe	526 (34.2)	104 (28.6)	236 (31.3)	113 (43.9)	43 (53.1)

Note: QoL-AD = quality of life in Alzheimer’s disease; VAS = visual analogue scale.

The average HRQoL score was 71.93 (s.d. 18.61), with an average QoL-AD score of 36.77 (s.d. 5.92). Information on the EQ-5D domains is shown in Table 1, with moderate or severe difficulties reported with mobility (41.8%), self-care (17.2%), performance of usual activities (34%), pain (40.2%) and anxiety or depression (34%).

### Prevalence and combinations of co-morbid conditions

**Figure 1:**
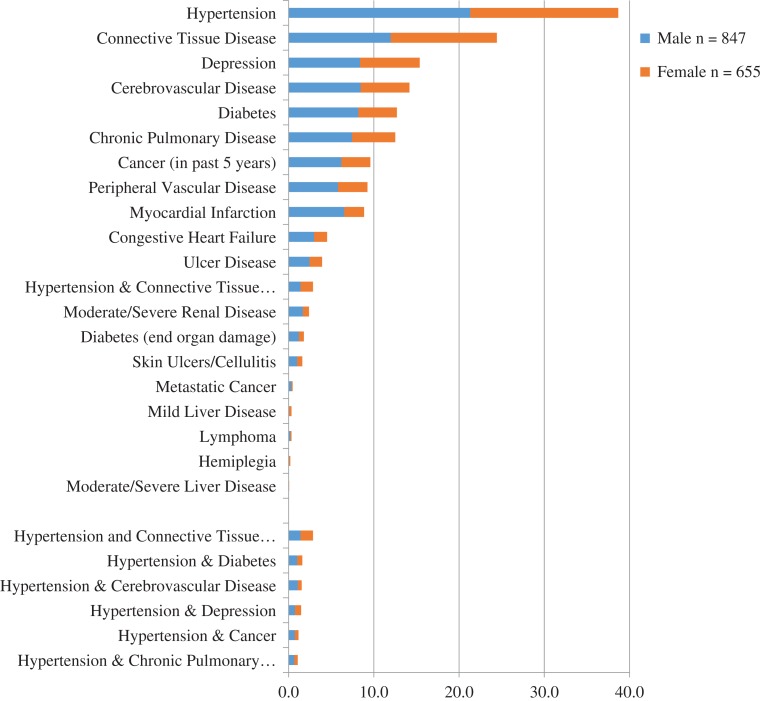
Prevalence of co-morbid conditions associated with dementia

The median number of co-morbid conditions reported was 1, with a range from 0 to 9. The prevalence of the individual co-morbid conditions included in the CCI for the whole sample and by gender is shown in Figure 1. The five most frequent conditions across the cohort were: hypertension (38.7%); connective tissue disease (24.5%); depression (15.4%); diabetes (12.7%); and chronic pulmonary disease (12.5%). This pattern of prevalence was similar in both males and females with the exception of the higher prevalence of myocardial infarction in men (11.6%) vs. 5.3% in women.

Over 300 combinations of co-morbid conditions were found but most combinations had low frequencies (*N* < 10). The most frequent two-way co-morbidities were hypertension with connective tissue disease (*N* = 43), hypertension and diabetes (*N* = 24), and hypertension and depression (*N* = 22). Three-way co-morbid combinations follow a similar pattern with hypertension present in all three of the top combinations: hypertension, connective disease and diabetes (*N* = 12); hypertension, cardiovascular disease and connective tissue disease (*N* = 8); and myocardial infarction, hypertension and connective tissue disease (*N* = 7).

Table 1 reports the number of co-morbid conditions by sociodemographic factors. In total, 25% of the sample did not report the presence of any co-morbid condition. The majority (52%) had mild (1–2 conditions) co-morbidity and only 5% had severe co-morbidity (>5 conditions). The distribution was similar across gender and age groups but varied across dementia subtypes. The percentage of vascular dementia and mixed AD and VD subtypes increased with the severity of co-morbid conditions.

Table 2 reports the associations between level of co-morbidity and ratings on the QoL-AD and the EQ-5D. There was a negative association between severity of co-morbid conditions and both QoL-AD and HRQoL scores. Compared to those with no chronic conditions, participants with severe co-morbidity (≥5 conditions) had lower QoL-AD by 6 points (−5.84; 95% CI: −7.27, −4.41) and lower HRQoL scores by 18 points (−17.82; 95% CI: −22.23, −13.42). After adjusting for age, sex, dementia subtypes and education, the adjusted estimates were −5.07 (95% CI: −6.53, −3.62) for QoL-AD and −15.85 (95% CI: −20.36, −11.34) for HRQoL.

**Table 2.** Unadjusted and adjusted associations between co-morbidity, health-rated quality of life (EQ-5D) and quality of life (QoL-AD) and the five EQ-5D domains

	Unadjusted model	Adjusted model*
	Coeff. (95% CI)	Coeff. (95% CI)
QoL-AD		
1–2 Mild vs. none	−1.45 (−2.19, −0.70)	−1.35 (−2.09, −0.62)
3–4 Moderate vs. none	−4.16 (−5.13, −3.20)	−4.07 (−5.05, −3.09)
≥5 Severe vs. none	−5.84 (−7.27, −4.41)	−5.07 (−6.53, −3.62)

EQ-5D VAS		
1–2 Mild vs. none	−5.15 (−7.43, −2.87)	−4.85 (−7.13, −2.57)
3–4 Moderate vs. none	−11.14 (−14.04, −8.24)	−10.58 (−13.53, −7.63)
≥5 Severe vs. none	−17.82 (−22.23, −13.42)	−15.85 (−20.36, −11.34)

EQ-5D domains	OR (95% CI)	OR (95% CI)
EQ-5D mobility		
1–2 Mild vs. none	2.18 (1.64, 2.90)	1.98 (1.46, 2.68)
3–4 Moderate vs. none	5.27 (3.71, 7.48)	4.44 (3.06, 6.45)
≥5 Severe vs. none	16.14 (8.63, 30.18)	14.64 (7.54, 28.41)
EQ-5D self-care		
1–2 Mild vs. none	1.77 (1.18, 2.67)	1.85 (1.20, 2.87)
3–4 Moderate vs. none	3.86 (2.46, 6.05)	4.50 (2.75, 7.37)
≥5 Severe vs. none	6.89 (3.90, 12.18)	6.46 (3.44, 12.13)
EQ-5D usual activities		
1–2 Mild vs. none	1.33 (1.00, 1.78)	1.24 (0.90, 1.69)
3–4 Moderate vs. none	2.71 (1.92, 3.83)	2.44 (1.68, 3.55)
≥5 Severe vs. none	6.44 (3.82, 10.85)	5.77 (3.28, 10.15)
EQ-5D Pain/discomfort		
1–2 Mild vs. none	2.06 (1.55, 2.73)	1.97 (1.47, 2.64)
3–4 Moderate vs. none	3.60 (2.56, 5.08)	3.43 (2.38, 4.92)
>5 Severe vs. none	9.56 (5.46, 16.73)	8.51 (4.72, 15.36)
EQ-5D Anxiety/Depression		
1–2 Mild vs. none	1.13 (0.86, 1.49)	1.18 (0.89, 1.58)
3–4 Moderate vs. none	1.96 (1.40, 2.74)	2.17 (1.52, 3.10)
≥5 Severe vs. none	2.83 (1.72, 4.62)	2.81 (1.66, 4.74)

*Adjusted for age group, sex, dementia subtypes and education. Note: QoL-AD = quality of life in Alzheimer’s disease; VAS = visual analogue scale.

For the five EQ5D domains severe co-morbidity was also associated with higher odds of moderate/severe problems in mobility (OR = 14.64; 95% CI:7.54, 28.41), self-care issues (OR = 6.46; 95% CI: 3.44, 12.13), impairment in usual activities (OR = 5.77; 95% CI: 3.28, 10.15), pain/discomfort (OR = 8.51; 95% CI: 4.72, 15.36) and anxiety/depression (OR = 2.81; 95% CI: 1.66, 4.74) after adjustment for sociodemographic factors (Table 2).

## Discussion

Using dementia as the index disease, this study examined the co-occurrence and combinations of conditions in people with dementia living in the community. The majority were living with one or two chronic health conditions, with 5% reporting more than five co-morbidities. Hypertension was the most frequent co-morbid condition. The severity of co-morbid conditions was associated with lower both HRQoL and dementia-specific QoL. People with severe co-morbid conditions had higher odds of problems in mobility, self-care, managing usual activities, pain and mood. The IDEAL cohort had similar rates of co-morbidity as those previously reported for people living with dementia [6], with 74% of people reporting more than one condition. Prevalence rates for co-morbidity vary across living situation and stages of dementia [7]. Co-morbidities linked with dementia identified through medical records report than 92% live with more than one condition [2]. Differences in prevalence rates may be attributed to the broader range of conditions encompassed in reviews of medical records than those collected by self-report [3].

Co-morbidities most prevalent in the dementia cohort were hypertension, connective tissue disease, depression, diabetes and chronic pulmonary disease. Combinations of conditions included hypertension with connective tissue disease, diabetes and depression. Hypertension prevalence rates are high in this age group [24]. For the main co-morbidities we identified there are known associations between both Alzheimer’s disease and vascular dementia and hypertension [25], and diabetes is a risk factor for Alzheimer’s disease and dementia [26]. Depression in mid- to late life has been linked with an increased risk of dementia [27], and the prevalence of depression in people with Alzheimer’s disease is high [28].

People with multiple chronic conditions were at greater risk of health problems in mobility, self-care, usual activities, pain/discomfort and anxiety/depression compared with people living with dementia only. The severity of co-morbid conditions was negatively associated with global ratings of HRQoL, consistent with previous studies [9, 13], and with disease-specific assessment of QoL [15]. Previous research has considered the impact of co-morbidity in one QoL domain [9] and this study provides additional evidence that co-morbidity may have a substantial impact on a person’s subjective perception of ill health and perceptions of well-being related to dementia.

There are some limitations in the measurement of co-morbidity within the study. Reliance on patient-reported co-morbidity may have led to the under-reporting of co-morbid conditions and confirmation with medical records is recommended for future studies [29]. The CCI mainly considers physical health conditions, and depression is the only mental health condition included. Depression can have an independent negative impact on QOL as well as amplifying the impact of physical conditions on perceived QOL [5]. The CCI does not encompass other physical changes or challenges associated with ageing (e.g. falls or frailty) that may also impact on QoL. There is growing recognition of the importance of considering frailty in combination with co-morbidity [30], and this has also extended to clinical guidelines [18]. A longitudinal perspective on the impact of co-morbidity as dementia progresses would be useful, as the co-morbidity may differ across stages of dementia and new chronic conditions may arise.

Co-morbidity in dementia may have serious implications for well-being, and information on the nature of co-morbidity is needed to plan adequate support and interventions [17]. The issue of co-morbidity poses significant challenges for care provision [4]. Dementia may impact on decision-making and on the self-management of chronic conditions [7]. There has been a move to identify groups at whom interventions to deal with co-morbidity should be targeted; those at most risk are people who find it difficult to manage everyday activities, those who are frail, and those who receive care from multiple sources [18].

This study suggests that co-morbidity is an important risk factor for poor health and global well-being in people with dementia. It is evident that people with dementia are experiencing the impact of living with multiple diseases and there is need for greater support and intervention in care provision and planning. Care providers often focus on individual conditions and this study highlights the need for better care planning and the organisation of care provision to deal with multiple conditions in an integrated way. The presence of multiple conditions is likely to increase the burden on carers who support people with dementia and the implications of this additional responsibility for carers should also be considered.

## Data access

The research materials supporting this publication will be made accessible through the UK Data Archive (http://www.data-archive.ac.uk/) in 2019.
